# Unilateral Non-Hemorrhagic Adrenal Infarction in Pregnancy: Case Series and Literature Review

**DOI:** 10.3390/jcm12144855

**Published:** 2023-07-24

**Authors:** Nikolai Tschuertz, Patrick Kupczyk, Bernd Poetzsch, Ulrich Gembruch, Waltraut M. Merz

**Affiliations:** 1 Department of Obstetrics and Prenatal Medicine, University Hospital Bonn, 53127 Bonn, Germany; 2 Department of Diagnostic and Interventional Radiology, University Hospital Bonn, 53127 Bonn, Germany; 3 Institute of Experimental Hematology and Transfusion Medicine, University Hospital Bonn, 53127 Bonn, Germany

**Keywords:** adrenal gland, adrenal infarction, non-hemorrhagic adrenal infarction, adrenal thrombosis, ischemia, pregnancy, abdominal pain, MRI, management, endocrinology

## Abstract

To summarize the evidence on non-hemorrhagic adrenal infarction (NHAI) and identify questions arising in diagnosis and management, cases in the PubMed database were merged with our case series. A total of 92 publications were retrieved, 15 of which reported on NHAI during pregnancy. Including the four in our case series, 24 cases have been described so far. Severe, unremitting pain requiring opioid analgesia was the leading symptom, often combined with nausea and vomiting. Laboratory results were non-contributory in most cases. Diagnosis was established via MRI in nine cases (37.5%) and via CT in six (25%); nine patients (37.5%) underwent both investigations. Location was predominantly on the right side (n = 16, 66.7%). In addition to analgesia, anticoagulation with heparin was commenced in 18 cases (75%). When thrombophilia screening was performed, major thrombogenic polymorphisms were detected in six cases (33.3%). One woman developed signs of adrenal insufficiency. The reported perinatal outcome was unremarkable. Unilateral NHAI has emerged as a rare but important cause of severe abdominal pain in pregnancy. The threshold to perform an MRI in pregnant women with characteristic clinical findings should be low. To prevent fetal radiation exposure, diagnostic imaging via CT should be avoided. In addition to symptomatic treatment with opioid analgesia, initiation of anticoagulant treatment should be strongly considered.

## 1. Introduction

Abdominal pain during pregnancy can have various causes, and establishing the correct diagnosis is challenging for several reasons. First, symptoms may be caused by obstetric complications. Second, changes in the position of intra-abdominal organs make the clinical assessment difficult. Finally, pregnancy-associated changes of reference values for several laboratory tests need to be taken into consideration [1].

If diagnostic imaging is required, ultrasonography is the mode of choice. However, its application for deep-lying abdominal soft tissue structures is limited. Additionally, the expanding uterus reduces visibility and therefore the diagnostic yield.

In case ultrasonography fails to establish a diagnosis, CT (computed tomography) and MRI (magnetic resonance imaging) are available. Both methods are particularly suitable for the imaging of soft tissue structures within the abdomen and pelvis.

Low-dose abdominal CT is associated with fetal radiation of some 1.5–35 mGy; the corresponding dose for pelvic CT amounts to 10–50 mGy [2]. The exposure depends on the number and spacing of adjacent image sections. Dose and timing during pregnancy determine the effects of ionizing radiation on the embryo/fetus.

From conception to the early second trimester, the teratogenic effect of ionizing radiation is of concern. At this gestational age, the recommended maximum permissible radiation dose is 5 mSv [3]. Another issue of concern is the effect of ionizing radiation on the developing brain, particularly if exposure occurs between 8 and 15 weeks of gestation. The minimum dose which may contribute to the development of microcephaly and intellectual disability is estimated to be 60–310 mGy. Below a radiation exposure threshold of 50 mGy, fetal anomalies and growth restriction may not occur [2].

Last, the stochastic effects of ionizing radiation need to be considered. This pertains especially to carcinogenesis. Fetal radiation exposure of 10–20 mGy may increase the risk for the development of childhood cancer (hematologic malignancies in particular) 1.5- to 2-fold [3]. More recent investigations calculated a lower effect (excess risk of a 10 mGy fetal dose producing an excess risk of 1 in 1667 to 1 in 4545) [4]. Due to the inevitable fetal radiation exposition, CT examinations should be avoided during pregnancy whenever possible.

In case CT needs to be performed, the application of iodinated contrast media does not seem to have a negative impact on the developing fetus with respect to teratogenesis or mutagenesis. Likewise, negative effects on the fetal thyroid have not been detected [5] Compared to CT, data on exposure to MRI during pregnancy are reassuring. Concerns regarding teratogenesis and potential effects of heat and acoustics have been expressed, but deleterious effects have not been detected. This also applies to MRI examinations with ≤3 Tesla field strength [6].

MRI has therefore emerged as the diagnostic imaging procedure of choice during pregnancy if ultrasonographic investigations fail to establish a diagnosis [1,4,5]. However, this statement only applies for non-enhanced MRI. Gadolinium-based contrast agents are known to pass the placenta. Mutagenic effects are unlikely, but the persistence of dissociated-free gadolinium within the fetus may increase the risk of stillbirth and neonatal death as well as the development of rheumatologic, inflammatory, or infiltrative skin conditions [7]. In cases where MRI is performed during pregnancy, the application of gadolinium-based contrast agents should therefore be avoided.

Since the first publication of unilateral non-hemorrhagic adrenal infarction (NHAI) diagnosed via MRI [8], further reports have been published [8,9,10,11,12,13]. NHAI during pregnancy needs to be differentiated from an acute bilateral adrenal hemorrhage. The latter usually occurs in patients with severe infections, coagulopathy, or after physical trauma [14]. To date, epidemiologic data on NHAIs are lacking. Likewise, etiology and pathogenesis are unknown. The adrenal perfusion is characterized by a rich arterial supply and drainage by a singular central vein. Pregnancy-associated hypercoagulability and the effect of the expanding uterus on the intra- and retroperitoneal structures may contribute to the development of NHAI [9,10,11,15,16,17,18].

Stipulated by our own series of four cases of NHAI during pregnancy at our institution within the past three years, we performed a literature search to summarize the published evidence on NHAI and to identify and address questions arising in diagnosis and management.

## 2. Materials and Methods

Pregnant women who presented for care at our center, a level IV university hospital, between the years 2018 and 2023 in whom a diagnosis of NHAI was established were prospectively followed. Results of laboratory tests and other investigations were collected. We recorded details of the maternal treatment, the delivery, and newborn data.

A literature review was performed using PubMed database from PubMed inception (January 1996) through May 2023. The search was restricted to publications in English. The following search terms were applied: ((adrenal) AND (infarction) AND (pregnancy)). In addition, references from original papers were manually searched for relevant citations. The exposure for our review was NHAI in pregnancy. Inclusion criteria were an observational study design, and a report of the maternal and perinatal outcome. We excluded reviews, editorials, and letters without sufficient data. Studies describing adrenal hemorrhage were excluded.

The extracted information included author, publication year, number of women, obstetric and medical history, medication, symptoms, types of investigations and results, treatment, delivery, newborn outcome, and long-term outcome.

Standard methods of descriptive statistics (median, percentage) were applied.

## 3. Results

**Figure 1:**
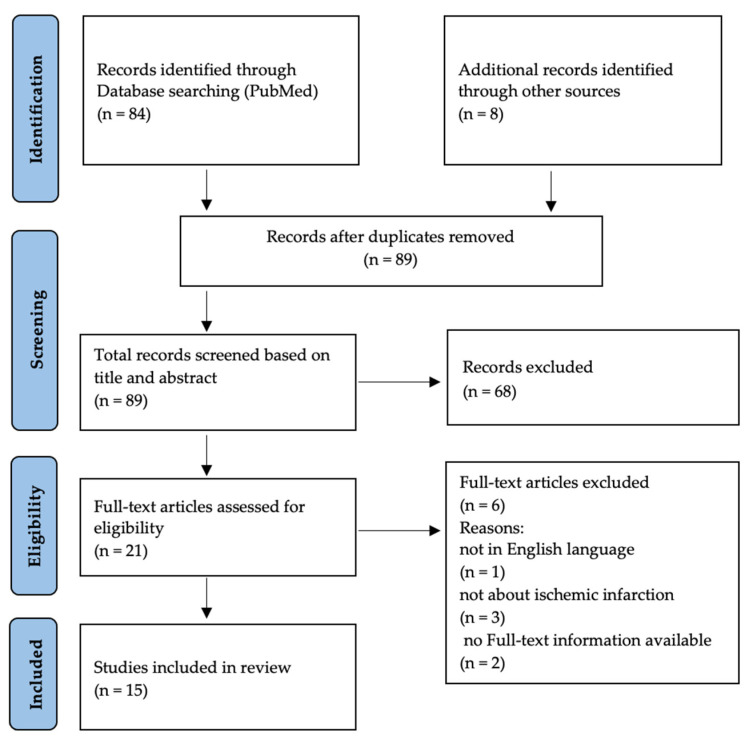
Flowchart: study identification, selection, and exclusions.

A total of 92 publications were retrieved, 15 of which reported on NHAI during pregnancy [8,9,10,11,12,13,15,16,19,20,21,22,23,24,25]. Only case reports and small case series were found. Figure 1 illustrates the identification, selection and exclusion process of our search. Including our own patients and excluding cases published twice [8,9,19,23,24,25], 24 cases are described [8,9,11,12,13,15,16,19,20,21,22,25].

**Figure 2:**
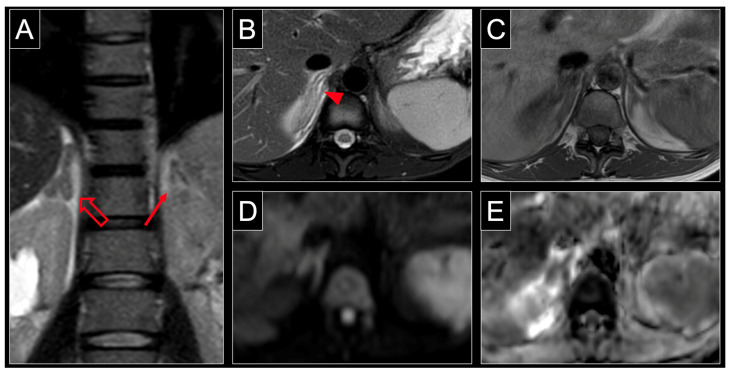
MRI of a 31-year-old patient at 34 weeks of pregnancy with non-hemorrhagic infarction of the right adrenal gland. Note the characteristic enlargement of the right adrenal gland (hollow arrow) compared to the normal contralateral adrenal gland (solid arrow) in the coronal plane of a T2 weighted sequence (**A**). Axial T2 weighting with fat saturation shows central and surrounding hyperintensity (arrowheads) reflecting organ edema with inflammatory response of retroperitoneal fat (**B**). The lack of intraparenchymal hyperintensity (arrowhead) in T1 weighting (**C**) indicates the absence of acute hemorrhage. Similar to other body regions, organ infarction is associated with restricted diffusion, which manifests as hyperintensity at high b-values, such as 800 s/mm^2^ (**D**) and low values in the ADC map (**E**) of DWI. ADC = apparent diffusion coefficient, DWI = diffusion weighted imaging.

Symptoms, investigations, management and outcome are listed in Table 1, Table 2 and Table 3. Typical MRI-findings are depicted in Figure 2 (case no 1, Table 1, Table 2 and Table 3) [10]. The median age was 29 years (IQR 24–31). No patient had a history of thromboembolic or ischemic events. The median gestational age (GA) at the onset of symptoms was 30 weeks of gestation (IQR 28–33). Laboratory results were non-contributory in most cases. Mildly increased markers of inflammation (leucocytosis, elevated C-reactive protein) or ketonuria were reported in eight cases (35%) (n = 3, 13% respectively).

Severe, unremitting pain requiring opioid analgesia was the leading symptom. The type of analgesia was not mentioned in three patients; one woman received an epidural analgesia. Diagnosis was established via MRI in nine cases and via CT in six; nine patients underwent both investigations. Location was predominantly on the right side (n = 16, 66.7%). In two cases (8.3%). both adrenal glands were affected. In addition to analgesia, 18 cases (75%) received anticoagulation with heparin in various dosages. Thrombophilia screening was performed in 18 cases (75%); major thrombogenic polymorphisms were detected in six cases (33.3%) (Factor V Leiden Mutation n = 2, Lupus anticoagulant n = 1, Methylenetetrahydrofolate (MTHFR) mutation n = 3).

Screening for adrenal insufficiency was performed in 17 cases (70.8%), predominantly postpartum (n = 11, 64.7%). One woman developed signs of adrenal insufficiency and required substitution. Of the reported perinatal outcomes, all were unremarkable. The majority of women (n = 14, 58.3%) received postpartum anticoagulation, with heparin being the preferred drug.

## 4. Discussion

We summarized existing evidence on unilateral NHAI during pregnancy. We retrieved only 24 cases with our literature search. Therefore, it seems to be an extremely rare condition. The reluctance to perform diagnostic imaging during pregnancy, and abdominal CT in particular, along with the steady increase of publications with the advent of high-resolution MRI leads us to suggest that that NHAI has gone and still may go unnoticed in a number of cases. The fact that the course of the disease is characterized by improvement and even remission with symptomatic therapy further lends support to this assumption. The high number of cases diagnosed at our institution (n = 4, 16.7% of all published cases) may be a result of a low threshold to perform non-enhanced MRI in pregnant symptomatic women if ultrasound and laboratory tests fail to establish a diagnosis.

Increased diagnostic yield associated with the widespread utilization of MRI may allow for a more detailed understanding of the pathophysiology and help to establish an evidence-based treatment approach. The sequence of events resulting in NHAI is not yet fully elucidated [9,10,11,15,16,17,18]. Based on the peculiarity of the adrenal perfusion with a rich arterial supply and a singular central vein an initial venous thrombotic event—either microvascular or of the adrenal vein—is favoured. This initial thrombotic event may be followed either by hemorrhage during reperfusion or by bypass of the thrombosed vessel(s) without hemorrhage. A spasm of the cortical arteries resulting in ischemic necrosis is another suggested pathomechanism. Pregnancy-induced hypercoagulability increases the risk of venous thromboembolism and thrombophilic risk factors further increase that risk. An initial thrombotic event is therefore the favoured pathomechanism. Accordingly, thrombophilia screening was performed in the majority of cases, showing a positive screening result in 6 out of 18 patients (33.3%).

The majority of events occurred on the right side. However bilateral NHAI occurred in two cases (8.3%). Preference of the right side may be the consequence of the anatomic features of the right adrenal vein: compared to the left adrenal vein it is very short and thin and enters the inferior vena cava directly and from dorsolaterally, thus increasing the chance of venous stasis [26,27]. A higher pressure of the gravid uterus on the retroperitoneal vessels, particularly with advanced gestational age (GA) further increases the chance of venous stasis. The advanced GA at the onset of symptoms supports our assumption.

No other risk factors were reported. Larger numbers are required to identify specific risk factors.

Due to the assumption of an initial thrombotic event initiation of anticoagulation is consistent. Various dosage and durations were chosen. With the impending delivery where hemorrhage is a major concern this question requires further attention. Dosage and duration of anticoagulation and/or antiaggregation need to be analyzed, aiming to avoid both, over- and undertreatment.

Only one case of adrenal insufficiency was reported. The necessity of screening for adrenal insufficiency on a regular basis, particularly in asymptomatic individuals, needs to be called into question.

Limitations of our study result from the small number of reported cases which precludes the calculation of prevalence and incidence. Likewise, recommendations regarding treatment and follow-up are based on very limited evidence.

## 5. Conclusions

In conclusion, unilateral NHAI has emerged as a rare but important cause of severe abdominal pain in pregnancy. The threshold to perform MRI in pregnant women with characteristic clinical findings should be low. Diagnostic imaging by CT should be abandoned to avoid fetal radiation exposure. In addition to symptomatic treatment with opioid analgesia, initiation of anticoagulant treatment at a therapeutic dosage should be strongly considered.

More data are required to identify risk factors, determine adequate treatment, and decide on endocrine follow-up. For a better understanding of this excruciating condition an international registry may be beneficial.

## Figures and Tables

**Table 1:** Overview: patient-related characteristics and history.

Author	Case No	Age	Parity	GA	Obstetric History	Medical History	Medication
Tschuertz et al.	1	31	1	33 4/7	1 NVD	Gall stones, nicotine, alcohol, cannabis, amphetamine abuse	quetiapine
Tschuertz et al.	2	26	1	29 4/7	1 NVD	unremarkable	none
Tschuertz et al.	3	27	3	32 2/7	3 NVD	gallstones	none
Tschuertz et al.	4	30	0	32 3/7	unremarkable	FVL (heterozygous), Hashimoto’s thyroiditis	L-Thyroxin
Agarwal 2019 [11]	5	21	0	28 4/7	unremarkable	asthma	none
Aljenaee 2017 [19]	6	29	4	24	4 NVD	unremarkable	none
Chague 2021 [10]	7	33	3	37	3 NVD	no personal/family hx of clotting disorders	nr
Chague 2021 [10]	8	38	1	26	1 NVD, 1 miscarriage	kidney stone, no personal/family hx of clotting disorders	nr
Chague 2021 [10]	9	19	0	32	twin pregnancy	L-sided pyelonephritis earlier during gestation, no personal/family hx of clotting disorders	nr
Chague 2021 [10]	10	34	0	31	unremarkable	no personal/family hx of clotting disorders	nr
Chague 2021 [10]	11	31	0	36	2 miscarriages	no personal/family hx of clotting disorders	nr
Chague 2021 [10]	12	22	0	30	unremarkable	no personal/family hx of clotting disorders	nr
Chasseloup 2019 [15]	13	30	3	30	2 NVD, 1 stillbirth	unremarkable	nr
Glomski 2018 [9] & Guenette 2015 [8]	14	20	0	27 4/7	unremarkable	no personal/family hx of clotting disorders	nr
Glomski 2018 [9]	15	24	nr	33	nr	no personal/family hx of clotting disorders	nr
Glomski 2018 [9] & Guenette 2015 [8]	16	29	1	17 & 35	unremarkable	no personal/family hx of clotting disorders	nr
Glomski 2018 [9]	17	33	nr	16	nr	no personal/family hx of clotting disorders	nr
Green 2013 [20]	18	25	0	28	cervical insufficiency, cerclage (2 miscarriages)	unremarkable	nr
Jerbaka 2021 [22]	19	36	7	36 5/7	7 NVD, 2 miscarriages	unremarkable	nr
Moliere 2017 [13]	20	29	1	30	1 NVD	nr	nr
Reichmann 2016 [12]	21	28	2	28 1/7	unremarkable	unremarkable	nr
Shah 2021 [21]	22	25	0	32	unremarkable	unremarkable	none
Sormunen-Harju 2016 [16]	23	31	1	38	1 NVD	unremarkable	none
Warda 2021 [25]	24	24	nr	30	nr	nr	nr

DVT: deep vein thrombosis, FVL: Factor V Leiden; GA: gestational age; hx: history; nr: not reported, NVD: normal vaginal delivery.

**Table 2:** Overview: symptoms and diagnostic procedures.

Author	Case No	Main Complaint	Pain Intensity	Investigations ^1^	Diagnostic Imaging Modality	Diagnostic Imaging Findings	Diagnosis
Tschuertz et al.	1	RUQ, R flank pain	8/10	WBC 14 G/L, CRP 7 mg/L. Ketonuria	MRI °	MRI: typical findings with fluid collection	R NHAI
Tschuertz et al.	2	R flank pain (3 days)	9/10	WBC 14.6 G/L, CRP 9.9 mg/L, RBC 9.7 g/dl, proteinuria (+), ketonuria	MRI °	MRI: typical findings	R NHAI
Tschuertz et al.	3	RUQ, R Flank pain	8/10	WBC 16.4 G/L CRP 5.9 mg/L, ketonuria, R hydronephrosis III	MRI °	MRI: typical findings with fluid collection	R NHAI
Tschuertz et al.	4	RUQ, RLQ, R Flank pain, N/V	9/10	WBC 20 G/L, CRP 16 mg/L, leukocyturia (++)	MRI °	MRI: typical findings with fluid collection	R NHAI
Agarwal 2019 [11]	5	RUQ pain 1/52, RLQ pain, N/V	nr	WBC 13.5 G/L, US: Murphy sign +, gall bladder sludge	MRI °, contrast-enhanced CT §	MRI: typical findings with fluid collection CT: typical findings	R NHAI
Aljenaee 2017 [19]	6	RUQ pain, N/V	severe	tachycardia, tachypnoea, WBC 10 G/L	low-dose contrast-enhanced CT §	CT: typical findings	R NHAI
Chague 2021 [10]	7	R-sided abdominal pain	nr	WBC 19 G/L, CRP 49 mg/L, D-dimer: 1070 ng/mL, US: R adrenal gland swelling	MRI °, contrast-enhanced CT §	MRI: typical findings, without any diffusion imaging CT: typical findings	R NHAI
Chague 2021 [10]	8	R flank pain	nr	WBC 20 G/L, CRP 17 mg/L	contrast-enhanced CT §	CT: typical findings with vein thrombus	R NHAI
Chague 2021 [10]	9	R flank pain	nr	WBC 18 G/L, CRP 82 mg/L, US: Pyelocaliceal dilatation and kidney stones	MRI °, contrast-enhanced CT §	CT: typical findings with vein thrombus MRI: typical findings	R NHAI
Chague 2021 [10]	10	RUQ pain	nr	WBC 15 G/L, CRP 25 mg/L, D-dimer 1500 ng/L	MRI °, contrast-enhanced CT §	CT: typical findings MRI: typical findings with fluid collection	R NHAI
Chague 2021 [10]	11	L-sided back pain, chest pain	nr	WBC 12.4 G/L, CRP 187 mg/L, D-dimer 820 ng/L, US: swelling L adrenal gland and fluid collection	MRI °, contrast-enhanced CT §	CT: typical findings MRI: typical findings with fluid collection	L NHAI
Chague 2021 [10]	12	L flank pain 1/7 later R flank pain	nr	WBC 10.3 G/L, CRP: 52 mg/L	MRI °, contrast-enhanced CT §	CT: bilateral typical findings with R vein thrombus MRI: bilateral typical findings	R + L NHAI
Chasseloup 2019 [15]	13	RUQ + back pain, contractions	nr	Biochemistry, ultrasound	low-dose contrast-enhanced CT ^2^	CT: R adrenal gland with typical findings. Both veins were enhancing	R NHAI
Glomski 2018 [9] & Guenette 2015 [8]	14	acute RUQ + R flank pain, N/V	severe	WBC 16.5 G/L, glucosuria (++), leukocyturia (+)	MRI °, low-dose contrast-enhanced CT §	MRI: retrospective slightly T2-hypointense R adrenal gland, diffusely enlarged CT: diffusely enlarged and hypoenhancing right adrenal gland with oedema	R NHAI
Glomski 2018 [9]	15	Acute LUQ pain, V, diarrhea	nr	WBC 13 G/L, L adrenal infarction (in retrospect)	MRI °	MRI: typical findings with fluid collection	L NHAI
Glomski 2018 [9] & Guenette 2015 [8]	16	1: acute pleuritic + RUQ + flank pain, N/V 2: acute L flank + epigastric pain, N	nr	1: WBC 13.5 G/L, R adrenal infarction (in retrospect) 2: WBC 15 G/L, L adrenal infarction	MRI °, low dose contrast-enhanced CT §	MRI: Uterine fibroid (17 + 5)-retrospectively (mildly enlarged adrenal gland, slightly hypointense on T2), perirenal fluid (35 + 5) CT in 35 + 5: L adrenal thickening, lack of enhancement	Uterine fibroid (17 + 5), L NHAI (35 + 5) (and R NHAI in retrospect)
Glomski 2018 [9]	17	persistent RLQ 1/52 after appendectomy, constipation	nr	WBC 11.4 G/L, R adrenal infarction (in retrospect)	MRI °	MRI: typical findings with fluid collection	R NHAI
Green 2013 [20]	18	acute RUQ and flank pain, N/V	nr	WBC 22.5 G/L	contrast-enhanced CT §	CT: typical findings	R NHAI
Jerbaka 2021 [22]	19	LUQ and L flank pain 2/7	nr	anemia, US: gall bladder sludge	after delivery: contrast-enhanced CT §	CT: decreased enhancement and adjacent inflammatory changes	L NHAI
Moliere 2017 [13]	20	epigastric pain, L + R flank + back pain, N	nr	bilateral adrenal ischemia	MRI °	MRI: bilateral enlargement with fluid collection	R + L adrenal ischemia
Reichmann 2016 [12]	21	acute R flank pain	intolerable	Biochemistry	MRI °	MRI: typical findings with fluid collection	R NHAI
Shah 2021 [21]	22	acute L flank pain, later sharp central lower chest pain, later R-sided abdominal pain	9/10	Lactate acidosis	contrast-enhanced CT §	CT: typical findings	L NHAI
Sormunen-Harju 2016 [16]	23	RUQ pain	nr	Proteinuria	after delivery: MRI with contrast, contrast-enhanced CT §	CT: typical findings with edema and thrombus MRI: typical findings with fluid collection and thrombus	initially preeclampsia suspected, thereafter R NHAI
Warda 2021 [25]	24	LUQ and back pain, N/V	nr	nr	MRI °	MRI: typical findings with fluid collection	L NHAI with necrosis

CRP: C-reactive protein; DVT: deep vein thrombosis; l: left; NHAI non-hemorrhagic adrenal infarction; nr: not reported; N/V: nausea and vomiting; r: right; RBC: red blood cell count; WBC: white blood cell count; °: no contrast fluid; §: radiation dosage not reported. ^1^ results within reference range not mentioned. ^2^ Omnipaque 350 contrast fluid (Dose-Length Product: 281 mGy/cm).

**Table 3:** Overview: treatment and outcome.

Author	Case No	Initial Treatment	Further Investigations ^1^	Further Treatment	Birth	Newborn	Postpartum Treatment	Long-Term Outcome
Tschuertz et al.	1	Opioids, Enoxaparin 60 mg bid 2/52	Holter ECG, TTE, thrombophilia and adrenal insufficiency screen	Enoxaparin 60 mg qd + ASS 100 mg qd until delivery	NVD 38 + 6	f, 2720 g, 8.P., Apgar * 8/9/10	Enoxaparin 60 mg qd 6/52, ASS if PFO	no FU
Tschuertz et al.	2	Opioids, antibiotics, enoxaparin 60 mg bid 2/52	TTE, thrombophilia screen (FVL heterozygous)	Enoxaparin 40 mg qd + ASS 100 qd until delivery	instrumental delivery 35 + 4	f, 2496 g, 36.P., Apgar * 7/9/10	Enoxaparin 40 mg qd 6/52, ASS 52/52, if PFO lifelong	no FU
Tschuertz et al.	3	opioids, antibiotics, enoxaparin 80 mg bid 2/52, thereafter 80 mg qd	thrombophilia screen (FVL heterozygous), adrenal insufficiency	Enoxaparin 80 mg qd, paused during labor	IoL, NVD 41 + 0	nr	Enoxaparin 40 mg qd 2/7, then 80 mg qd 6/52, ASS 100 mg qd lifelong	no FU
Tschuertz et al.	4	opioids, antibiotics, enoxaparin 80 mg bid 2/52, thereafter 40 mg qd, ASS 100 mg/d	TTE, Holter ECG, Duplex US	Enoxaparin 80 mg bid 2/52, 40 mg qd + ASS 100 mg qd until delivery	NVD 37 + 1	f, 2530 g 13.P., APGAR * 10/10/10	nr	no FU
Agarwal 2019 [11]	5	opioids, antibiotics, enoxaparin 80 mg bid, duration nr	Thrombophilia and adrenal insufficiency screen, bubble-TTE (PFO)	enoxaparin 80 mg bid until delivery, UFH during delivery	IoL, NVD 40+	nr, healthy	LMWH 6/52 and lifelong ASS recommendation (not done)	8/52 no adrenal insufficiency
Aljenaee 2017 [19]	6	LMWH bid, (therapeutic dose, duration nr)	thrombophilia and DVT screen	LMWH until delivery (dosage nr)	PROM 37+, NVD	nr, healthy	LMWH 2/52	2nd thrombophilia screen after 6/52 (FVIII elevation), confirmation 12/52 later, recommendation for lifelong anticoagulation, No adrenal insufficiency
Chague 2021 [10]	7	Opioids	thrombophilia screening	IoL	IoL, NVD	nr	OAK, ASS 48/52 (dosages nr)	CT 3/12 and 30/12: Atrophic adrenal with partially restored glandular enhancement
Chague 2021 [10]	8	Opioids, Heparin (dosage and duration nr)	thrombophilia screen	Heparin until delivery (dosage nr)	nr	nr	OAK 6/12 (dosage nr)	MRI 1/12, CT-enhanced 3/12: Atrophic adrenal with partially restored glandular enhancement, no adrenal insufficiency
Chague 2021 [10]	9	Opioids, Heparin (dosage and duration nr)	thrombophilia screen	Heparin (dosage and duration nr)	nr	nr	OAK 3/12 (dosage nr)	CT 3/12: isolated residual atrophy of the lateral arm of the gland
Chague 2021 [10]	10	Opioids, Heparin (dosage and duration nr)	thrombophilia screen	Heparin during pregnancy (dosage nr)	nr	nr	no anticoagulation	MRI 1/52: no change. MRI 4/52: Appearance of T1-weighted hyperintensity
Chague 2021 [10]	11	Opioids, Heparin (dosage and duration nr)	thrombophilia screen	Heparin during pregnancy (dosage nr)	nr	nr	no anticoagulation	MRI 3/12: swollen L adrenal gland. Collection decreased with partially restored glandular enhancement
Chague 2021 [10]	12	Opioids, Heparin (dosage and duration nr)	thrombophilia screen (LA+)	Heparin, Hydrocortisone during pregnancy (dosage nr)	nr	nr	no anticoagulation	nr
Chasseloup 2019 [15]	13	opioids, nifedipine, RDS prophylaxis (betamethasone)	nr	nr	NVD	healthy, preterm 2500 g	enoxaparin bid 26/52	ACTH Test, thrombophilia screen, CT (R adrenal atrophy)
Glomski 2018 [9] & Guenette 2015 [8]	14	Opioids, heparin drip (dosage and duration nr)	thrombophilia screen	LMWH until delivery, dosage nr	CS 39 + 4, obstetric indication	healthy, 3150 g	nr	CT 3 years later for suspected PE: normalization of the R adrenal gland, no adrenal insufficiency
Glomski 2018 [9]	15	Opioids	nr	nr	NVD 37+	healthy	nr	no adrenal insufficiency
Glomski 2018 [9] & Guenette 2015 [8]	16	1: Opioids, NSAR. 2: Opioids, heparin drip (dosage and duration nr)	thrombophilia screen	1: Opioid analgesia, NSAR 2: Opioid analgesia, heparin drip (dosage and duration nr)	CS GA 36 due to inadequate pain control	healthy, 3200 g, APGAR 6/8/X	LMWH (dosage and duration nr)	MRI and CT Scan 12/52 (second NHAI) normal R adrenal gland. no adrenal insufficiency
Glomski 2018 [9]	17	Opioids	nr	nr	IoL, NVD 40+	healthy	nr	no adrenal insufficiency
Green 2013 [20]	18	enoxaparin 80 mg bid (duration nr)	ACTH, thrombophilia screen (MTHFR heterozygous)	nr	PPROM, NVD 33+	liveborn, 2180 g, APGAR * 9/9/X	LMWH bid 6/52	no FU
Jerbaka 2021 [22]	19	Spasmolytic, PPI	ACTH Test, thrombophilia screen (MTHFR C677T homozygous, HPA1 1a/1b heterozygous)	IoL	IoL, NVD	male, 3040 g APGAR * 9/10/nr	LMWH 40 qd 1/7, LMWH 60 bid 7/7, LMWH 60 qd 6/12	6/12: no adrenal insufficiency
Moliere 2017 [13]	20	Heparin (dosage and duration nr)	nr	Heparin during the pregnancy (dosage nr)	nr	nr	nr	no adrenal insufficiency
Reichmann 2016 [12]	21	Opioids, LMWH 60 mg bid (duration nr)	thrombophilia screen	LMWH until delivery (dosage nr)	nr	nr	nr	no adrenal insufficiency
Shah 2021 [21]	22	opioids, antibiotics	nr	post-op hypotensive, adrenal insufficiency 3d post-op (hydrocortisone + warfarin)	PPROM, CS 32+	liveborn, 1000 g, NICU	warfarin 12/52 (dosage nr)	thrombophilia screen, 12/52 later: SST with adrenal insufficiency hydrocortisone continued, warfarin stopped, 7/12 later: Normal SST
Sormunen-Harju 2016 [16]	23	Magnesium Sulfate, IoL, epidural analgesia	thrombophilia and adrenal insufficiency screening	nr	IoL, NVD	3415 g, male	LMWH 60 bid 12/52, ASS (dosage nr)	ASS for 1–2 years, 4/52 MRI: thrombosis and edema subsided. 12/52: adrenal atrophy, no adrenal insufficiency
Warda 2021 [25]	24	Opioids, LMWH bid (dosage and duration nr)	ACTH Test, thrombophilia screen (MTHFR A1298C heterozygous)	Hydrocortisone (stress dose, discharged on physiologic dose), LMWH qd (dosage nr)	NVD 39+ with Hydrocortisone (stress dose)	healthy, f	Hydrocortisone qd, LMWH nr	nr

APGAR: * at 1/5/10 min respectively; ASS: acetyl salicylic acid; bid: twice per day; CS: cesarean section, ECG: electrocardiogram; f: female; FU: follow-up; FVL: factor V Leiden; IoL: induction of labor; LA: lupus anticoagulant; LMWH: low molecular weight heparin; m: male; NICU: neonatal intensive care unit; nr: not reported; NVD: normal vaginal delivery; OAK: oral anticoagulant; P.: percentile; PFO: persistent foramen ovale; PPROM: preterm premature rupture of membranes; PROM: premature rupture of membranes; qd: once per day; RDS: respiratory distress syndrome; SST: short synacthen test; TTE: transthoracic echocardiography. ^1^ only abnormal result specified.

## Data Availability

The data sets used and/or analyzed during the current study are available from the corresponding author on reasonable request.
